# Effect of Sex and Prior Exposure to a Cafeteria Diet on the Distribution of Sex Hormones between Plasma and Blood Cells

**DOI:** 10.1371/journal.pone.0034381

**Published:** 2012-03-27

**Authors:** María del Mar Romero, José Antonio Fernández-López, Xavier Remesar, Marià Alemany

**Affiliations:** 1 Department of Nutrition and Food Science, Faculty of Biology, University of Barcelona, Barcelona, Spain; 2 Institute of Biomedicine, University of Barcelona, Barcelona, Spain; 3 CIBER (Centro de Investigación Biomédica en Red) Physiopathology of Obesity and Nutrition, National Institute of Health ‘Carlos III’, Madrid, Spain; Hosptial Infantil Universitario Niño Jesús, CIBEROBN, Spain

## Abstract

It is generally assumed that steroid hormones are carried in the blood free and/or bound to plasma proteins. We investigated whether blood cells were also able to bind/carry sex-related hormones: estrone, estradiol, DHEA and testosterone. Wistar male and female rats were fed a cafeteria diet for 30 days, which induced overweight. The rats were fed the standard rat diet for 15 additional days to minimize the immediate effects of excess ingested energy. Controls were always kept on standard diet. After the rats were killed, their blood was used for 1) measuring plasma hormone levels, 2) determining the binding of labeled hormones to washed red blood cells (RBC), 3) incubating whole blood with labeled hormones and determining the distribution of label between plasma and packed cells, discounting the trapped plasma volume, 4) determining free plasma hormone using labeled hormones, both through membrane ultrafiltration and dextran-charcoal removal. The results were computed individually for each rat. Cells retained up to 32% estrone, and down to 10% of testosterone, with marked differences due to sex and diet (the latter only for estrogens, not for DHEA and testosterone). Sex and diet also affected the concentrations of all hormones, with no significant diet effects for estradiol and DHEA, but with considerable interaction between both factors. Binding to RBC was non-specific for all hormones. Estrogen distribution in plasma compartments was affected by sex and diet. In conclusion: a) there is a large non-specific RBC-carried compartment for estrone, estradiol, DHEA and testosterone deeply affected by sex; b) Prior exposure to a cafeteria (hyperlipidic) diet induced hormone distribution changes, affected by sex, which hint at sex-related structural differences in RBC membranes; c) We postulate that the RBC compartment may contribute to maintain free (i.e., fully active) sex hormone levels in a way similar to plasma proteins non-specific binding.

## Introduction

Steroid hormones are found free in blood plasma, but a variable proportion of these hormones is carried bound to protein, in a few cases with high affinity, as is the case of SHBG (Sex Hormone-Binding Globulin) [1] in humans, binding both 17β-estradiol and testosterone, or CBG (Corticosteroid-Binding Globulin) [2], essentially binding cortisol/corticosterone but also testosterone to a lower extent [3]. There are a number of other steroid binding proteins in plasma [4], but other proteins also bind non-specifically these hormones with a varying degree of affinity [5]; i.e. the large amount of albumin in plasma may be responsible for a considerable proportion of this binding [6] not because of high affinity but simply because of its bulk.

There are clues of the possible intervention of bound hormones or the globulins themselves as signals [7]–[9], since it is generally assumed that only the “free” hormones [10] are ready to bind the specific hormone receptors on the cell surface. However, the existence of membrane-related hormone-binding globulins, as is the case of CBG [8], [11] suggest that precisely the high affinity of these hormone transporters may help facilitate the crossing of the hormone into the cell and/or the binding of the hormone to its receptor [12].

Red blood cells (RBC) constitute somewhat less than half the volume of blood, and are responsible for most of its viscosity and cross the capillary beds normally with a tight fitting. It has been found that there is a direct interchange of a few chemical substances between the RBC surface and that of the endothelial lining [13]–[16]. Other molecules, such as amino acids (and probably glucose) are loosely bound to the red blood cell surface, favoring a rapid interchange with the capillary endothelial cells [13].

The possible role of RBC in the transport of steroid hormones was advanced soon after most of these hormones were identified [17], [18]. Their relative structural similarity to cholesterol and usually high lipophilic nature help reinforce this assumption. In fact there is a number of studies describing the association of steroid hormones [18], [19] and cell membranes. The direct biophysical interaction of some hormones, such as estradiol and membranes has been also analyzed [20].

The existence of a RBC-surface “compartment” for small molecular weight metabolites, the lipophilic nature of most steroid hormones and the particular direct cell-to-cell contact of RBC and the capillary bed lining made us to decide to investigate whether RBC may be a quantitatively significant hormone blood compartment, as well as to set the bases for the analysis of its possible direct relationship with the other blood hormone compartments, now simplified into: a) free, b) plasma protein-bound, and c) RBC-carried. We wanted to determine the effects of sex and mild obesity, thus we used a 30-day exposure to a cafeteria diet, but delayed the utilization of these animals for 15 days in order to minimize the eventual immediate effects of the diet, but retain the medium-to-long term effects of incipient phase of metabolic syndrome development [21], [22].

## Results

Table 1 show the weights of rats at the beginning of the study, after 30 days (i.e. at the end of cafeteria diet treatment) and two weeks later, when they were killed. Females were smaller than males in all cases. Cafeteria diet increased more the weight of treated animals than controls on normal rat chow, as shown by the significant differences on day 30. However, a slight rise of weight in controls during the 2-week normalisation period (statistically significant for females) and a slight decrease in those of cafeteria (not significant) eliminated the significance of the effect of diet on the final weight. There were no effects of sex or diet in protein percentage with respect to body weight, neither of sex for lipids, but exposure to cafeteria diet resulted in significantly higher percentages of body lipids.

**Table 1:** Body weight changes, food intake, body composition and hematocrit of rats; effects of sex and prior exposure to a cafeteria diet.

	units	female		male		p values		
		control	cafeteria	control	cafeteria	sex	diet	int.
Initial body weight (day 0)	g	190±5	202±6	291±3	298±6	<0.0001	NS	NS
body weight (day 30, end of cafeteria diet)	g	229±5○	271±4○	362±8○	425±10○	<0.0001	<0.0001	NS
final body weight (day 45)	g	250±6○ •	261±7○	399±11○	417±10○	<0.0001	NS	NS
body composition: lipids	% BW	13.1±1.1	15.7±1.5	12.9±1.0	15.6±1.4	NS	0.0492	NS
body composition: protein	% BW	19.6±0.6	19.2±1.1	19.1±1.3	18.7±0.9	NS	NS	NS
mean food energy intake (days 0–30)	kJ/day	276±15	605±28	368±19	680±39	<0.0001	<0.0001	<0.0001
mean food energy intake (days 30–45)	kJ/day	265±11	221±10*	365±14	326±11*	<0.0001	0.0019	NS
hematocrit	%	41.4±0.7	42.1±0.4	40.3±0.5	46.1±1.4	0.0010	NS	0.0068

N = 6 in all groups. %BW = percentage of body weight. Statistical comparisons between groups (two-way ANOVA: sex, diet and interaction [int.]): NS represents a p>0.05. Comparison of weight and food intake at different times in the same groups; a symbol represents a significant p<0.05 difference, Student's t test):

○ = weight different vs. day 0;

• = weight different between days 30 and 45;

* daily food intake different between days 0–30 and 30–45.

Food energy intake was higher in males than in females. During cafeteria diet feeding, the rats exposed to this diet ingested a mean daily energy intake 2.2-fold higher in females and 1.9-fold higher in males than controls fed the standard chow. After removal of cafeteria diet, in the next 15 days, ex-cafeteria rats energy intake was slightly (albeit significantly) lower than that of controls, both for male and female rats.

The use of a transient (30 days followed by 15 days of dietary normalization) exposure to a cafeteria diet was used as a way to limit the immediate response of hormones to diet, but nevertheless leaving the imprint of a fattening period in the animals: we were looking for lasting effects on blood hormone compartmentation not immediately attributable to diet (but obviously dependent on its past and lasting effects). There were no diet-related significant effects of diet on plasma levels of glucose, triacylglycerols, cholesterol, total plasma proteins and urea in these animals (results not shown). There were, however, significant differences (effect of sex, but not of diet) in hematocrit between the four experimental groups (Table 1); the interaction diet-sex was also significant: the (delayed) response to diet was higher in male than in female rats.

The distribution of trace tritum-labeled sex hormones in the blood was significantly affected by sex in all cases, and by exposure to a cafeteria diet only in the case of estrogens: estrone and estradiol (Table 2); in both cases there was also a significant interaction on the effects of sex and diet, the effects of diet being more marked in males. Binding of the hormones to RBC was higher in males than in females, and was not uniform. In control rats, both males and females, there were significant differences in the percentage of label present in RBC for all four hormones tested. The maximal percentage of cell-carried hormone was that of estrone, followed by estradiol, DHEA and testosterone, the latter about one half or less of the percentage of carried estrone. Temporal exposure to cafeteria diet modified slightly the situation, so that now there were no differences between the percentages of labeled estradiol and DHEA bound to blood cells.

**Table 2:** Distribution of tritium hormone in the blood cells of rats, effects of sex and prior exposure to a cafeteria diet.

	female		male		p values		
	control	cafeteria	control	cafeteria	sex	diet	int.
estrone	24.3±1.0^A^	24.1±0.7^A^	31.6±1.0^A^	26.3±0.4^A^	<0.0001	0.0300	0.0052
estradiol	19.6±1.0^B^	17.6±0.3^B^	28.5±0.6^B^	22.2±0.9^B^	<0.0001	<0.0001	0.0097
DHEA	16.4±0.4^C^	17.5±0.3^B^	20.8±0.7^C^	21.3±0.4^B^	<0.0001	NS	NS
testosterone	9.9±0.8^D^	12.1±0.4^C^	15.5±0.6^D^	16.0±0.9^C^	<0.0001	NS	NS

The data represent the percentage of hormone bound to blood cells. The label corresponding to the plasma trapped in packed blood cells (3.5% in volume of packed cells) has been discounted from the data presented. N = 6 in all groups.

Statistical comparisons between groups (two-way ANOVA: sex, diet and interaction [int.]): NS represents a p>0.05. The differences in percentages of labelled hormone for each group of rats (column) were statistically significant as a whole (one way-ANOVA, p<0.0001 in all cases). Different superscript letters correspond to statistically significant differences between hormones (post-hoc analysis, p<0.05) in the same rat group.

The analysis of binding of labeled hormones to fresh RBC suspensions showed no significant specific binding to RBC of any of the four hormones tested (data not shown).

Table 3 presents the measured plasma concentrations of estrone, estradiol, DHEA and testosterone as well as the estimated total blood concentration of the same hormones individually calculated for each rat from the plasma data and the cell bound hormone data in Table 2. In all cases, the effect of sex was statistically significant, with higher estrogens and DHEA, and lower testosterone plasma levels in females. The effect of transient cafeteria diet exposure was also significant for estrone and testosterone, but not for estradiol or DHEA. The blood values followed the same pattern. There were, again, significant interactions between sex and diet for estrone, DHEA and testosterone (only total blood, not plasma): the effects of cafeteria diet exposure were influenced by sex in all hormones except estradiol. The blood data were significantly lower than those of plasma for all groups.

**Table 3:** Concentrations of hormones in the plasma (and blood) of rats, effects of sex and prior exposure to a cafeteria diet.

	units	sample	female		male		p values		
			control	cafeteria	control	cafeteria	sex	diet	int.
estrone	pM	plasma	186±24	487±48	129±15	234±23	<0.0001	<0.0001	0.0040
		blood	148±18	380±36	116±14	176±18	0.0001	<0.0001	0.0014
estradiol	pM	plasma	49.6±17.4	47.1±6.2	18.8±2.8	22.7±2.1	0.0082	NS	NS
		blood	36.7±12.3	33.9±4.0	16.1±2.4	16.1±0.7	0.0087	NS	NS
DHEA	nM	plasma	2.45±0.11	2.95±0.14	2.12±0.20	1.33±0.44	0.0012	NS	0.0210
		blood	1.76±0.08	2.12±0.10	1.64±0.17	0.93±0.12	<0.0001	NS	0.0003
testosterone	nM	plasma	7.47±0.49	5.01±0.31	37.9±4.4	25.7±2.5	<0.0001	0.0093	NS
		blood	4.98±0.37	3.38±0.20	27.3±3.0	16.9±1.5	<0.0001	0.0020	0.0170

The data for plasma were directly measured using RIA or ELISA procedures, for blood levels estimation see the text. N = 6 in all cases.

Statistical comparisons between groups (two-way ANOVA: sex, diet and interaction [int.]): NS represents a p>0.05. In all groups, the differences between plasma and blood values were statistically significant (p<0.05; paired Student's t test, p<0.05).

**Figure 1:**
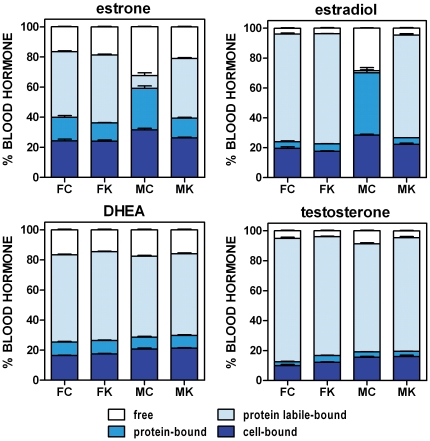
Percent distribution of sex-related hormones in blood compartments of male and female rats previously subjected to a cafeteria diet. Experimental groups: FC = female control; FK = female cafeteria; MC = male control; MK = male cafeteria. Statistical differences between groups (2-way ANOVA): The effects of “sex” and “diet” were statistically significant (p<0.05) for all hormones and blood compartments except: DHEA (*diet* in cells and both protein fractions, and *sex* in protein-bound hormone) and testosterone (*diet* in cells and protein labile-bound hormone, and *sex* in protein-bound hormone).

**Figure 2:**
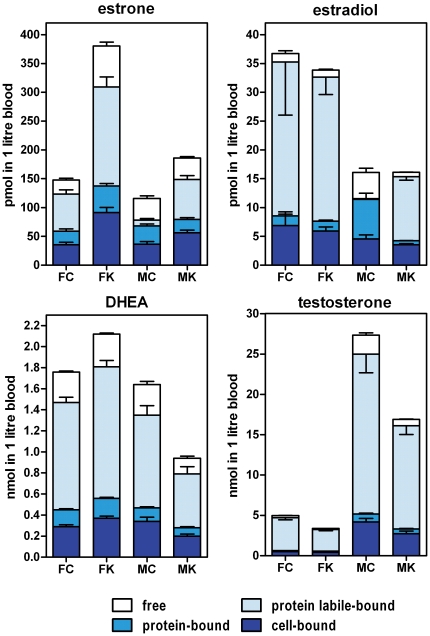
Blood compartment distribution of sex hormones in rats, effect of sex and previous exposure to a cafeteria diet. Experimental groups: FC = female control; FK = female cafeteria; MC = male control; MK = male cafeteria. Statistical differences between groups (2-way ANOVA). The effects of “sex” and “diet” were statistically significant (p<0.05) for all hormones and blood compartments except: estrone (*sex* and *diet* in protein-bound hormone), estradiol (*diet* in cell-bound and protein-labile bound hormone), DHEA (*diet* in cell-bound, protein-bound and free hormone).

The compartmentation of sex-related hormones in rat blood is shown in Figure 1 (percentage distribution in the different blood fractions) and Figure 2 (molar concentration values). The patterns of distribution of the different hormones studied were different (Figure 1), and, especially for estrogens, there were marked sex and diet differences. In contrast, DHEA percentage distribution was fairly uniform, as was that of testosterone to a certain degree. The differences were more marked when the actual hormone levels were taken into account (Figure 2).

Under the experimental conditions tested, most of the four hormones can be found either free or loosely bound to proteins, with a smaller fraction bound more strongly to plasma proteins.

Control male estrone levels were only 78% than those of females, but free estrone was almost twice, these differences were the consequence of differences in plasma protein-bound hormone; labile-bound hormone in males was only 16% of the corresponding values of females. However, cafeteria diet corrected most of these differences (in percentage), increasing total estrone by 1.5× in males and 2.6× in females.

Estradiol levels were lower in males (less than half of the female values), and were little affected by cafeteria diet, however, the distribution in compartments followed closely that described for estrone in control males, with a high proportion of free estradiol and almost negligible labile-protein bound hormone.

DHEA levels in male controls were 93% of those of females. The distribution in compartments was similar for all four groups. Cafeteria diet induced a relative increase in total blood DHEA (×1.2) but in males the effect was the opposite, a drop of −43%.

Testosterone levels were higher in male controls (×5.5) than those of females, the differences being even higher for free hormone (×9.5); cafeteria diet decreased total testosterone in both sexes in a similar proportion (−32% in females and −38% in males), but free testosterone decreased even higher: −48% in females and −68% in males.

## Discussion

The presence of steroid hormones in RBC has been known for a time [17], and the possibility of their binding [23], [24], transport [19] or implication in membrane structure [20], [25] have been already advanced by different groups of investigators. However, the extent and possible equilibrium role of the RBC-bound hormone compartment has not been –to our knowledge— investigated, and neither have the effects of diet and sex. The main consequence of the present study is the finding that there are marked sexual differences in the ability of RBC to carry sex hormones, which strongly hints at different membrane structural differences. Furthermore, the previous exposure to an obesogenic diet strongly modulates these effects, which points to the RBC (membrane) as a possible hormone carrier or circulating reservoir.

The large amounts of hormones (and the fast interchange between related molecular species) found in packed cells, as well as the small size [26] and precise analysis of trapped plasma, exclude the possibility that this trapped plasma be the sole source of the hormones found in the cells compartment. Its contribution is in any case in the range of 5–10% of total packed-cell hormone, and its significance has been measured and taken into account in the calculations.

We did not establish in which stage of the estrous cycle were the rats used in the study, but the levels of estradiol were in the range of rats in diestrus [27], their uniform intragroup values suggest that all were in the same estral phase. We don't know whether the estral cycle changes may affect rat estradiol compartmentation in a way similar to their changes in concentration [27], [28].

A few minutes (data not shown; we routinely used 120 minutes as standard incubation time for the sake of saturation) of incubation of plasma with tracer amounts of labeled hormones, were sufficient for interchange with the cold hormone molecules (or, either, induce additional binding). This suggests that there may be an actual interchange, and eventually equilibrium, between plasma- and cell-carried hormone molecules, in line with those described for glucose and amino acids [13], [29]. The low sem values with respect to the means for hormone distribution data, also suggests the absence of external interfering factors. In any case, the relative uniformity in the proportions of hormone distributed in the three main compartments of the blood (Table 2, Figure 1) in spite of its dependence on sex, diet and concentration suggests that there exists an equilibrium between these compartments, and, consequently, that RBC-carried hormones may also participate in the eventual supply of free hormones to bind to their targets, acting as buffering reserve in a way not different from that of non-specific binding to proteins. In fact, this compartment has a potentially higher absolute binding/storage capacity than plasma proteins and probably is a main buffering site for the maintenance of stabilized hormone plasma levels.

Blood cells carry a significant proportion of sex hormones, as previously observed for cortisol [17] and estrogens [19], in a way that simple plasma/serum analyses leave behind a significant proportion (about 1/4 or more) of blood hormone. It may be suggested that RBC-carried hormones, because of their binding to the cells should be less dynamic than the free hormone or that bound non-specifically to most plasma proteins, in closer contact with the free hormone. However, the results presented here suggest otherwise: RBC-carried hormones may behave just as another (albeit larger) non-specific hormone carrier compartment in blood, not different in this aspect from unspecified plasma proteins. In addition, most of the present-day circulating hormone analyses precisely include the sum of the plasma protein-bound and free hormone compartments as a single unit for reference, comparative and diagnostic values.

Since rat plasma does not contain SHBG as humans do [30], [31], we can safely assume that most of the protein-bound testosterone and estradiol is non-specific [5], [32]. The use of charcoal-dextran may have partially displaced the proportions of protein-bound versus loosely bound plus free hormone in the plasma fraction. This method is widely used for separation of free hormones [33], [34], but the use of a more specific free-hormone separation system [34] allowed us to better discriminate between these two entities: loosely bound hormone and free hormone. However, both methods are routinely used to measure “free” hormone levels. The differences observed here clearly suggest that their application is not discriminative enough (the results for the same blood sample and hormone are systematically different). This is a question that should be taken into account by clinicians and investigators measuring serum hormone levels, and is a source of variability (and perhaps of endocrine significance) which is more than often overlooked, in a similar way to the compartment we have analyzed and brought to scrutiny: the blood cell hormone compartment. This, in itself may be also modulated by packed cell volume, but more probably by a different membrane affinity for steroid hormones, a consequence of their structure or, more probably of the previous binding of other steroids, such as cholesterol [19] or, more probably, estradiol itself [18], which may help explain the sex-related differences in the size of the RBC compartment for all the hormones studied.

In any case, the distribution presented here is in line with the concept of equilibrium/continuity of free and loosely-bound hormone as a continuum of physiological significance; the model also includes the hormone bound to proteins in the plasma (specific or not) which freeing is less immediate. We suggest that this continuum should also include the RBC. This may be especially important in the pass of RBC across capillary beds with the corresponding cell-to-cell contact and transfer of materials [14].

The binding of the four hormones to isolated RBC showed that the binding was non-specific, i.e. not linked to specific sites, which agrees with the known interaction of estradiol with plasma membranes [19], [20] and the lipophilic nature of the four hormones selected. Probably, the mechanism of loose-binding of the hormones to membranes is in part comparable to that of cholesterol [19], [35]. The occasional presence of fatty acid-esterified sex hormones in blood (especially in lipoproteins) [36]–[38] has been attributed to their similarity with cholesterol, resulting in their trans-esterification [39].

The differences between hormones on their binding to RBC could not be solely attributed to their different lipophilia, since testosterone is more lipophilic than DHEA and the latter is not much different from estradiol. Nevertheless, the most lipophilic of the four, estrone, is the hormone bound in larger amounts by RBC. The lipophilic explanation, however, does not fully hold when we compare the significant and maintained differences between male and female RBC, males binding a higher proportion of label (versus plasma). This may be a consequence of different membrane structure of RBC depending on sex, but there is no published information backing the existence of these differences, in fact blood transfusions do not take into account the sex of the donor. Hyperlipidic diets modify the lipid composition of plasma membranes, including those of RBC [40], [41], and higher exposure to glucose and oxidative agents linked to the metabolic syndrome, obesity and hyperlipidic diets [42] also provoke changes in RBC membrane fluidity and cell deformability [43], [44]. Males are more sensitive to these changes and show earlier (and with deeper intensity) the consequences of hyperlipidic diets [45], [46] as a harbinger of the development of the metabolic syndrome [47]. A 30-day exposure to a cafeteria diet (a normal carbohydrate and protein content, but hyperlipidic diet [48]) may change the membrane structure and thus affect hormone binding. The data presented here show that the male-female differences tend to decrease with prior exposure to the diet, and the distribution of hormones into blood compartments is more uniform.

The deep differences in plasma compartment distribution of estrogens between male and female controls suggest that the specificity of hormone binding proteins in plasma may be considerably altered by sex, in a way that overall energy availability may also modulate this distribution. The patterns observed for the MC group for estradiol and estrone (the analyses were repeated three times), and the low overall variability of the data suggest that sex modulation of protein-binding of estrogens is a possibility that merits further analysis.

We can only speculate on the significance of the RBC hormone compartment, but also on the sex-related differences in its size or capability. Susceptibility to hyperlipidic diets is higher in males than in females [45], [46], a condition that has been related to the antioxidant and anti-inflammatory effects of estrogen in endothelia and at the cell membrane level [39], [49], Why should the RBC membranes be different from the endothelial ones in this respect? Both face oxidative aggression in the same milieu, the capillary beds. A higher abundance of estradiol in RBC membranes may change their fluidity and capacity to be altered by oxidation or nitration.

In spite of the decrease in sex-related differences induced by prior exposure to cafeteria diet on the carrying of sex hormones by blood cells, the effect of sex persists as a clear differentiating factor for most hormones (DHEA being the sole, albeit partial, exception). This may be explained only as a difference in membrane structure between male- and female-derived cells. The repeated existence of interactions between the effects of sex and diet and even the different direction of the changes induced by diet on females and males are consequent with a different carrying ability of the RBC membranes. The more marked response elicited by estrogen also agrees with their higher affinity for membranes [20], [35], the higher binding observed here and the protecting and antioxidant function of estrogens at the cell membrane level [39], [49], which has been put forward as a main factor in the overall protective effects of estrogen against the ravages of the metabolic syndrome [15], [16].

The different response of the four hormones tested to the exposure to a cafeteria diet also indicate that in spite of the binding not being specific, changes in membrane composition, structure or ability to react (long-term given the long half-life of RBCs) may affect the differential binding of different molecular species, thus affecting the postulated role of secondary hormone buffer-carrier of RBC. The data presented suggest that the RBC hormone compartment is largely a correlate of total blood-carried hormone, which also helps reinforce the postulated buffering role of this compartment.

The existence of an additional plasma reservoir of sex hormones may help to stabilize their levels, and is closely related to their medium-term effects, requiring less brusque changes in comparison with peptide hormones and many cytokines (or catecholamines, eliciting immediate responses). More detailed analyses are needed before speculating further on the physiological role of this compartment. So far, the only question that is crystal clear is its existence, which has an immediate methodological consequence: the actual and functional concentrations of sex hormones in blood may not be a direct correlate of their plasma levels, in the same way that free plasma (or serum) hormone levels are not a direct correlate of total hormone plasma levels.

In conclusion, we have found that there is a large non-specific RBC-carried compartment in the blood which contains a sizeable proportion of estrone, estradiol, DHEA and testosterone; there are marked and significant differences between sexes as to the size of these pools. Prior exposure to a cafeteria (hyperlipidic) diet induced some changes, clearly modulated or influenced by sex, which hint at structural differences in RBC membrane structure or composition. It is suggested that the RBC compartment may contribute to the maintenance of free (i.e. fully active) sex hormone levels in a way comparable, and probably complementary, to their (mainly non-specific) binding to plasma proteins.

## Materials and Methods

### Ethics statement

All animal handling procedures were done in accordance with the norms of European, Spanish and Catalan Governments. The study was specifically approved (DMAH-5483) by the Animal Ethics Committee of the University of Barcelona.

### Animals and animal handling

Wistar adult rats (9 week-old) both male and female were used (Harlan Laboratories Models, Sant Feliu de Codines, Spain). The rats were adapted to the Animal House environment for at least 7 days prior to the beginning of the experiment, and were fed the standard Harlan (type 2014) chow. Half of the rats in each group were subjected to an energy-rich limited-item cafeteria diet [21] for a month, followed by 15 days of standard rat chow (2014 Harlan), whilst the other groups were kept as controls eating all the time the usual rat chow (45 days). Food consumption and rat weights were recorded. The rats were kept in adjoining collective 3-rat cages.

The four experimental groups (N = 6 for each) were: female-control, female-cafeteria, male-control and male-cafeteria. A group of untreated rats, the same age as controls was used for the estimation of the trapped plasma volume in packed cells. The rats were killed in complete groups in five consecutive days (female controls, male controls, male cafeteria and female cafeteria), i.e. the time between female rat killings was the equivalent to a 4-day estrous cycle.

At the end of the experiment (i.e. on day 45), the rats were anaesthetized with isoflurane and killed by exsanguination (aortic blood drawing with a dry-heparinized syringe). Part of the blood was immediately centrifuged (at 600×g for 10 minutes at 2–4°C). Plasma was then centrifuged for 10 additional minutes at 3000×g at 2–4°C. Plasma was frozen and kept at −20°C. Packed cells were gently resuspended in phosphate-buffered saline (PBS) pH 7.4 (12 mM phosphate buffer containing 140 mM NaCl), washed three times and used for hormone-binding assays. A second aliquot of blood was used fresh for the analysis of labelled hormone distribution.

Body composition was estimated by freezing the dead rats, autoclaving them in sealed bags and then homogenizing the remains. After thorough mixing, samples of rat paste were used for total lipid extraction with trichloromethane: methanol [50], and semiautomated Kjeldahl total N analysis using a ProNitro S semiautomatic system (JP Selecta, Abrera, Spain). Body N was converted to total protein by using a rat-specific equivalence factor [51].

### Trapped plasma volume in centrifuged packed cells

In a series of experiments, the volume of trapped plasma in the packed cell pellet [26] was established by using freshly extracted blood to which tracer amounts (20 kBq/ml) of ^14^C-labelled sucrose (PerkinElmer, Rodgau, Germany) were added. The blood was gently mixed and then subjected to centrifugation, ranging from 1000×g to 16000×g during 5 to 30 minutes at 2–4°C. In each case, the radioactivity of the supernatant plasma and that of packed cells were measured (N = 5). The presence of label in the pellet was translated to volume of trapped plasma, since sucrose does not bind to red blood cells but is freely dissolved in plasma.

**Figure 3:**
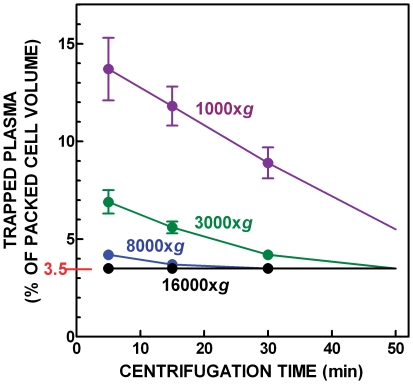
Proportion of trapped plasma in rat blood packed cell volume with increasing time and speed of centrifugation.

The lowest cell breakage with minimal trapped plasma (or supernatant) volume were established at 8000–16000×g, for 10–20 minutes, which gave a mean trapped plasma volume of 3.5±0.0%. Since there was very little variation over this value with changes in time-speed (Figure 3), the final conditions used were 16000×g centrifugation for 20 minutes at 2–4°C and a standard trapped plasma volume of 3.5% was used for all ensuing calculations. These data were correlated with hematocrit values in order to use this latter value to estimate the trapped plasma in packed RBC.

### Hormone binding to RBC

Fresh blood cells resuspended in PBS were used for affinity binding assays using labeled hormones at different concentrations (from 20 pM to 100 nM) in 0.2 ml RBC suspension (i.e. about 40 mg of cells). They were incubated for 120 min at 20°C. The incubation was stopped by setting the tubes in an ice bath and immediately filtering-out the cells under vacuum through glass fiber filters (GFC Whatman, Maidstone, Kent UK). The filters were used to measure the label retained by the RBCs through scintillation counting. The addition of 200-fold cold hormone concentration in half the samples was used to differentiate specific from non-specific binding [52]. No specific binding by whole RBCs was observed in any sample.

All labeled hormones were obtained from PerkinElmer, Boston, MA USA). Label and specific activity of the products used were: (2,4,6,7-^3^H) estrone (2.4 MBq/nmol), (2,4,6,7,16,17-^3^H) 17β-estradiol (6.0 MBq/nmol), (1,2,6,7-^3^H) dehydroepiandrosterone (2.7 MBq/nmol), and (1,2,6,7-^3^H) testosterone (2.6 MBq/nmol). Cold hormones were obtained from Sigma (St Louis, MO USA).

### Distribution of labeled hormones in blood

Aliquots (0.25 ml) of fresh blood were introduced, in triplicate, in Eppendorf vials; then, labeled hormones (carrier-free) diluted in buffer were added to each vial (5 µl, i.e. 3.7 kBq) using the same pipette tip for all samples of the same hormone and for the measurement of the introduced label. The tubes were left 2 hours in a gently shaking bath at 37°C; then were centrifuged at 16000×g for 20 min in the cold. Samples of supernatant plasma and packed cells were obtained and their total weight (used to calculate their volume) and radioactivity were measured. The results were corrected for the trapped plasma volume as indicated above.

Two complementary methods to estimate the free hormone levels were used: treatment with charcoal-dextran [53] and ultrafiltration [54].

A sample was treated, again in triplicate, with charcoal-dextran [53] for 5 minutes under gentle shaking, and then centrifuged 5 min at 2000×g at 2–4°C. The label remaining in the supernatant (plasma) was measured. Another sample was centrifuged, using Centrifree tubes (Millipore Ireland, Carrigywohill, Ireland), at 1500×g for 20 minutes at 30°C, and the ultrafiltrate radioactivity was also measured.

Differences in whole plasma, plasma washed-out with charcoal-dextran and plasma ultrafiltrate data for each rat were use to establish the distribution of label (i.e. hormone) in plasma in three main “compartments”. Free hormone in ultrafiltrates was always smaller than that retained by charcoal-dextran, and was assumed to correspond to the actually free hormone. The difference between this value and that retained (precipitated) by charcoal-dextran was assumed to correspond to hormone feebly bound to proteins, i.e. labile binding; the label retained in plasma proteins in spite of charcoal-dextran exposure was assumed to correspond to hormone more strongly bound to proteins, i.e. truly protein-bound hormone.

### Measurement of the plasma levels of hormones

Plasma samples were used for analysis of hormone levels in duplicate. Estrone was measured using an ELISA kit (DB52051, IBL International, Hamburg, Germany), 17β-estradiol with a ultra-sensitive estradiol RIA (DBL-4800, Beckman-Coulter, Brea CA USA), dehydroepiandrosterone with a RIA (DRG, Mountanside, NJ USA), and testosterone with an ELISA kit (RE52151, IBL International). Hormone analyses were done following the instructions of the manufacturers.

### Calculations

Our working hypothesis assumed that in whole blood there are at least three main steroid hormone compartments: free, bound to plasma proteins, and bound to RBC. So that for a given volume, total blood hormone was the sum of the three compartments; and plasma hormone was that measured in plasma plus the fraction “t” trapped in packed cells. There is a complex mixture of more or less-specifically binding proteins, and the concept of “free” is also imprecise because it depends on exposure and differential binding force of carrier proteins and tissue membranes/receptors. In any case the data were useful for comparative analysis since we used all experimentally-derived data from each individual rat.

Plasma hormone concentration could be accurately measured by direct RIA or ELISA, and the proportions of free and protein-bound hormone (in two subfractions: protein-bound and protein-labile binding) can be subsequently calculated from the charcoal-dextran data and ultrafiltration label distribution. The presence of hormone in RBC was estimated discounting the trapped-plasma contribution (i.e. 3.5% of packed cell volume corresponding to plasma, which hormone concentration we had measured). The data of distribution of tracer between plasma and packed cells was a remarkably constant value for each hormone. Thus we used this quotient to estimate the amount of label present in packed cells, and from that, and the label corresponding to trapped plasma, calculate the label retained in the RBC.

Since the ratio of RBC versus plasma label distribution “r” was known, the label in RBC would be H·P·r·(1−t·H), where “P” was the plasma hormone concentration, “t” was the proportion of trapped plasma (i.e. 0.035) and “H” was the hematocrit fraction (Hc/100). In consequence, the contribution of plasma to total blood hormone would be the sum of the hormone found in plasma plus that trapped in packed cells: (1−H)·[P·(1−r)+(t·H)].

Since all these parameters (P, H, r) were estimated for each individual rat and t variation was negligible, we could estimate the blood hormone concentration for each rat of the four hormones studied. The proportion of hormone in each of the three compartments (free, plasma-bound and cell-bound) was also calculated for each individual rat.

Statistical analysis was carried out using one- and two-way ANOVA analysis (sex, diet) with the Newman-Keuls post-hoc test, as well as the paired Student's *t* test, using the Statgraphics Centurion XVI program package (Statpoint Technologies, Warrengton VA USA).

## References

[1] Mickelson KE, Pétra PH (1975). Purification of the sex steroid binding protein from human serum.. Biochemistry.

[2] Chader GJ, Westphal U (1968). Steroid-protein interactions. XVI. Isolation and characterization of the corticosteroid-binding globulin of the rabbit.. J Biol Chem.

[3] Pearlman WH, Crépy O (1967). Steroid-protein interaction with particular reference to testosterone binding by human serum.. J Biol Chem.

[4] Egleston BL, Chandler DW, Dorgan JF (2010). Validity of estimating non-sex hormone-binding globulin bound testosterone and oestradiol from total hormone measurements in boys and girls.. Ann Clin Biochem.

[5] Lea OA, Støa KF (1972). The binding of testosterone to different serum proteins: A comparative study.. J Steroid Biochem.

[6] Pardridge WM, Mietus LJ (1979). Transport of steroid hormones through the rat blood-brain barrier. Primary role of albumin-bound hormone.. J Clin Invest.

[7] Bonnet F, Balkau B, Malécot JM, Picard P, Lange C (2009). Sex hormone-binding globulin predicts the incidence of hyperglycemia in women: interactions with adiponectin levels.. Eur J Endocrinol.

[8] Kuhn RW (1988). Corticosteroid-binding globulin interactions with target cells and plasma membranes.. Ann NY Acad Sci.

[9] Vanbillemont G, Lapauw B, Bogaert V, Goemaere S, Zmierczak HG (2010). Sex hormone-binding globulin as an independent determinant of cortical bone status in men at the age of peak bone mass.. J Clin Endocrinol Metab.

[10] Hackbarth JS, Hoyne JB, Grebe SK, Singh RJ (2011). Accuracy of calculated free testosterone differs between equations and depends on gender and SHBG concentration.. Steroids.

[11] Ibarrola I, Andrés M, Marino A, Macarulla JM, Trueba M (1996). Purification of a cortisol binding protein from hepatic plasma membrane.. Biochim Biophys Acta.

[12] Grasa MM, Cabot C, Fernández-López JA, Remesar X, Alemany M (2001). Modulation of corticosterone availability to white adipose tissue of lean and obese Zucker rats by corticosteroid-binding globulin.. Horm Metabol Res.

[13] Proenza AM, Palou A, Roca P (1994). Amino acid distribution in human blood. A significant pool of amino acids is adsorbed onto blood cell membranes.. Biochem Mol Biol Int.

[14] Watts RP, Brendel K, Luthra MG, Kim HD (1979). Inosine from liver as a possible energy source for pig red blood cells.. Life Sci.

[15] Moolman JA (2006). Unravelling the cardioprotective mechanism of action of estrogens.. Cardiovasc Res.

[16] Xing DQ, Nozell S, Chen YF, Hage F, Oparil S (2009). Estrogen and mechanisms of vascular protection.. Arterioscler Thromb Vasc Biol.

[17] Farese RV, Plager JE (1962). The *in vitro* red blood cell uptake of C^14^-cortisol; studies of plasma protein binding of cortisol in normal and absnormal states.. J Clin Invest.

[18] Migeon CJ, Lescure OL, Zinkham WH, Sidbury JB (1962). *In vitro* interconversion of 16-C^14^-estrone and 16-C^14^-estradiol-17b by erythrocytes from normal subjects and from subjects with a deficiency of red cell glucose-6-phosphate dehydrogenase activity.. J Clin Invest.

[19] Jacobsohn MK, Bauder S, Pine SR, Jacobsohn GM (1994). Cholesterol limits estrogen uptake by liposomes and erythrocyte membranes.. Biochim Biophys Acta.

[20] Scheidt HA, Badeau RM, Huster D (2010). Investigating the membrane orientation and transversal distribution of 17β-estradiol in lipid membranes by solid-state NMR.. Chem Phys Lipids.

[21] Ferrer-Lorente R, Cabot C, Fernández-López JA, Alemany M (2005). Combined effects of oleoyl-estrone and a β_3_-adrenergic agonist (CL316,243) on lipid stores of diet-induced overweight male Wistar rats.. Life Sci.

[22] Romero MM, Esteve M, Alemany M (2006). Combined effects of oral oleoyl-estrone and limited food intake on body composition of young overweight male rats.. Int J Obesity.

[23] Doucet DR, Bonitz RP, Feinman R, Colorado I, Ramanathan M (2010). Estrogenic hormone modulation abrogates changes in red blood cell deformability and neutrophil activation in trauma hemorrhagic shock.. J Trauma-Inj Infect Crit Care.

[24] Hiramatsu R, Nisula BC (1988). Erythrocyte-associated component of blood cortisol.. Ann NY Acad Sci.

[25] LePetit-Thevenin J, Lerique B, Nobili O, Boyer J (1991). Estrogen modulates phospholipid acylation in red blood cells: relationship to cell aging.. Am J Physiol.

[26] Ferrando A, Alemany M, Bobadilla IG, Bobadilla MS, Palou A (1981). Comparative estimation of hematocrit and trapped plasma in the packed cell-volume in man, rabbit and chicken blood.. Comp Biochem Physiol A.

[27] Nequin LG, Alvarez J, Schwartz NB (1979). Measurement of serum steroid and gonadotropin levels and uterine and ovarian variables throughout 4 day and 5 day estrous cycles in the rat.. Biol Reprod.

[28] Geiger JM, Plas-Roser S, Aron C (1980). Mechanisms of ovulation of female rats treated with FSH at the beginning of the estrous cycle: Changes in pituitary responsiveness to luteinizing hormone releasing hormone (LHRH).. Biol Reprod.

[29] Palou A, Remesar X, Arola L, Alemany M (1980). Blood and plasma glucose relationships during pregnancy, the breeding cycle and development in the rat.. Diabete Metabol.

[30] Ritzen EM, Nayfeh SN, French FS, Dobbins MC (1971). Demonstration of androgen-binding components in rat epididymis cytosol and comparison with binding components in prostate and other tissues.. Endocrinology.

[31] Joseph DR, Hall SH, French FS (1985). Identification of complementary DNA clones that encode rat androgen binding protein.. J Androl.

[32] Södegard R, Bäckström T, Shanbhag V, Carstensen H (1982). Calculation of free and bound fractions of testosterone and estradiol-17β to human plasma proteins at body temperature.. J Steroid Biochem.

[33] Dufau ML, Catt KJ, Tsuruhara T, Ryan D (1972). Radioimmunoassay for plasma testosterone.. Clinica Chimica Acta.

[34] Vhen Y, Yazdanpanah M, Wang XY, Hoffman BR, Diamandis EP (2010). Direct measurement of serum free testosterone by ultrafiltration followed by liquid chromatography tandem mass spectrometry.. Clin Biochem.

[35] Peri A, Benvenuti S, Luciani P, Deledda C, Cellai I (2011). Membrane cholesterol as a mediator of the neuroprotective effects of estrogens.. Neurosci.

[36] Vihma V, Tikkanen MJ (2011). Fatty acid esters of steroids: Synthesis and metabolism in lipoproteins and adipose tissue.. J Steroid Biochem Mol Biol.

[37] Höckerstedt A, Jauhiainen M, Tikkanen MJ (2006). Estradiol fatty acid esterification is increased in high density lipoprotein subclass 3 isolated from hypertriglyceridemic subjects.. Atherosclerosis.

[38] Leszczynski DE, Schafer RM, Perkins EG, Jerrell JP, Kummerow FA (1989). Esterificaction of dehydroepiandrosterone by human plasma HDL_3_.. Biochim Biophys Acta.

[39] Höckerstedt A, Jauhiainen M, Tikkanen MJ (2004). Lecithin/cholesterol acyltransferase induces estradiol esterification in high-density lipoprotein, increasing its antioxidant potential.. J Clin Endocrinol Metab.

[40] Katan MB, Deslypere JP, van Birgelen AP, Penders M, Zegwaard M (1997). Kinetics of the incorporation of dietary fatty acids into serum cholesteryl esters, erythrocyte membranes, and adipose tissue: an 18-month controlled study.. J Lipid Res.

[41] Vidgren HM, Ågren JJ, Schwab U, Rissanen T, Hänninen O (1997). Incorporation of n-3 fatty acids into plasma lipid fractions, and erythrocyte membranes and platelets during dietary supplementation with fish, fish oil, and docosahexaenoic acid-rich oil among healthy young men.. Lipids.

[42] Roberts CK, Barnard RJ, Sindhu RK, Jurczak M, Ehdaie A (2006). Oxidative stress and dysregulation of NAD(P)H oxidase and antioxidant enzymes in diet-induced metabolic syndrome.. Metabolism.

[43] Garnier M, Attali JR, Valensi P, Delatour-Hanss E, Gaudey F (1990). Erythrocyte deformability in diabetes and erythrocyte membrane lipid composition.. Metabolism.

[44] Vayá A, Cámara R, Hernández-Mijares A, Romagnoli M, Solà E (2010). Erythrocyte deformability in morbid obesity before bariatric surgery. Influence of abdominal obesity.. Clin Hemorheol Microcircul.

[45] Tchoukalova YD, Koutsari C, Votruba SB, Tchkonia T, Giorgadze N (2010). Sex- and depot-dependent differences in adipogenesis in normal-weight humans.. Obesity.

[46] Lovejoy JC, Sainsbury A (2009). Sex differences in obesity and the regulation of energy homeostasis.. Obes Rev.

[47] Melanson EL, Astrup A, Donahoo WT (2009). The relationship between dietary fat and fatty acid intake and body weight, diabetes, and the metabolic syndrome.. Ann Nutr Metab.

[48] Prats E, Monfar M, Iglesias R, Castellà J, Alemany M (1989). Energy intake of rats fed a cafeteria diet.. Physiol Behav.

[49] Prokai L, Prokai-Tatrai K, Perjési P, Simpkins JW (2005). Mechanistic insights into the direct antioxidant effects of estrogens.. Drug Develop Res.

[50] Folch J, Lees M, Sloane-Stanley GH (1957). A simple method for the isolation and purification of total lipides from animal tissues.. J Biol Chem.

[51] Rafecas I, Esteve M, Fernández-López JA, Remesar X, Alemany M (1994). Whole rat protein content rstimation. Applicability of the Nx6.25 factor.. Br J Nutr.

[52] Davenport AP, Russell FD, Maher SJ (1996). Radioligand binding assays: Theory and practice.. Current directions in radiopharmaceutical research and development.

[53] Mendel CM, Miller MB, Siiteri PK, Murai JT (1990). Rates of dissociation of steroid and thyroid hormones from human serum albumin.. J Steroid Biochem Mol Biol.

[54] Ly LP, Handelsman DJ (2005). Empirical estimation of free testosterone and sex hormone-binding globulin immunoassays.. Eur J Endocrinol.

